# Detection of colonic neoplasia *in vivo* using near-infrared-labeled peptide targeting cMet

**DOI:** 10.1038/s41598-019-54385-7

**Published:** 2019-11-29

**Authors:** Xiaoli Wu, Juan Zhou, Fa Wang, Xiaoqing Meng, Jing Chen, Tse-Shao Chang, Miki Lee, Gaoming Li, Xue Li, Henry D. Appelman, Rork Kuick, Thomas D. Wang

**Affiliations:** 1 Division of Gastroenterology, Department of Internal Medicine, University of Michigan, Ann Arbor, Michigan USA; 2 Department of Pathology, University of Michigan, Ann Arbor, Michigan USA; 3 Department of Biostatistics, University of Michigan, Ann Arbor, Michigan USA; 4 Department of Biomedical Engineering, University of Michigan, Ann Arbor, Michigan USA; 5 Department of Mechanical Engineering, University of Michigan, Ann Arbor, Michigan USA

**Keywords:** Cancer imaging, Cancer imaging, Colon cancer, Colon cancer

## Abstract

White light colonoscopy is widely used to detect colorectal polyps, but flat and depressed lesions are often missed. Here, we report a molecular imaging strategy to potentially improve diagnostic performance by developing a fluorescently-labeled peptide specific for cMet. This 7mer is conjugated to Cy5.5, a near-infrared (NIR) cyanine dye. Specific binding to cMet was confirmed by cell staining, knockdown, and competition assays. The probe showed high binding affinity (k_d_ = 57 nM) and fast onset (k = 1.6 min) to support topical administration *in vivo*. A mouse model (*CPC;Apc*) that develops spontaneous adenomas that overexpress cMet was used to demonstrate feasibility for real time *in vivo* imaging. This targeting ligand showed significantly higher target-to-background (T/B) ratio for polypoid and non-polypoid lesions by comparison with a scrambled control peptide. Immunofluorescence staining on human colon specimens show significantly greater binding to tubular and sessile serrated adenomas versus hyperplastic polyps and normal mucosa. These results demonstrate a peptide specific for cMet that is promising for endoscopic detection of pre-malignant lesions and guiding of tissue biopsy.

## Introduction

Colorectal cancer (CRC) is a leading cause of cancer-related morbidity and mortality worldwide^1,2^, thus improved methods for early CRC detection are needed. Conventional white light colonoscopy is recommended for patients at increased risk, and is used to identify structural changes^3^. However, the miss rate for grossly visible polyps can be over 25%^4–8^, and pre-malignant lesions can be flat in appearance and easily missed^9–12^. Interval cancers occur when CRC arises within 5 years after a colonoscopy exam, and are increasing in incidence^13–15^. Methods of advanced imaging are being developed to improve performance for early CRC detection. Chromoendoscopy uses topically-administered intravital dyes and narrow band imaging (NBI) uses filtered light in different spectral bands to highlight mucosal changes suspicious for disease^16,17^. In these approaches, contrast is generated from non-specific mechanisms that are unrelated to the biological processes that drive CRC progression, and have shown limited effectiveness in clinical studies.

cMet is expressed on the surface of normal epithelial cells in the digestive tract, and is highly overexpressed in CRC^18–20^. Recently, cMet has been found to be overexpressed in pre-malignant colonic lesions, thus is a promising target for early cancer detection^21^. The cMet oncogene encodes a transmembrane tyrosine kinase receptor^22,23^. This 190 kD heterodimer consists of two subunits linked by disulfide bonds, including an extra-cellular 50 kD α-chain and a transmembrane 145 kD β-chain. Hepatocyte growth factor (HGF) binds to cMet to trigger autophosphorylatation and activation of downstream mitogen-activated protein kinase (MAPK), phosphatidylinositol 3-kinase (PI3K), and transcription (STAT) signaling^24^. This pathway plays an important role in tumor growth, invasion, angiogenesis, and metastasis^25^. In addition to colon, cMet is overexpressed in other cancers, including pancreatic, gastric, hepatocellular, breast, and sarcoma^26–28^.

Peptides are promising for clinical use as ligands to detect molecular targets that are specific for pre-malignant lesions. They can bind to cell surface targets with high specificity and affinity on the nanomolar scale^29–32^. With topical application, peptides can be delivered effectively to mucosal surfaces in the digestive tract at high concentrations to maximize target interactions and achieve rapid binding with minimal risk for toxicity^33,34^. This probe platform has flexibility to be labeled with a broad range of fluorophores^35^, and is inexpensive to mass manufacture^36^. Peptides have low potential for immunogenicity, allowing for repeat use^37,38^. These features of peptides are well suited for clinical use in high volume procedures, such as colonoscopy. Here, we aim to identify and validate a peptide specific for cMet, and demonstrate *in vivo* use to detect pre-malignant colonic lesions that are flat in appearance and can be easily missed by white light illumination.

## Results

### Peptide specific for cMet

**Figure 1:**
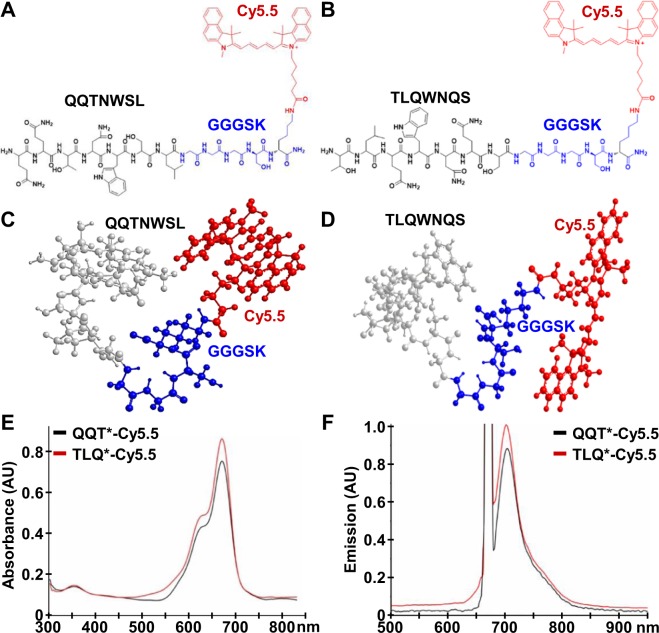
Peptide specific for cMet. (**A**) QQTNWSL (black) is labeled with a Cy5.5 fluorophore (red) via a GGGSK linker (blue). (**B**) Sequence is scrambled as TLQWNQS for control. (**C**,**D**) 3D models show differences in biochemical structures. (**E**) Peak absorbance occurs at λ_abs_ = 675 nm. (**F**) Maximum emission with λ_ex_ = 671 nm excitation occurs at λ_em_ = 710 nm.

Phage display was used to biopan a linear heptapeptide library against the extra-cellular domain (ECD) of cMet. QQTNWSL showed the lowest *P*-value for binding interactions using a structural model for cMet. The C-terminus of this peptide was covalently linked with a Cy5.5 fluorophore via a GGGSK linker, hereafter QQT*-Cy5.5, Fig. 1A. The linker separates the peptide from the fluorophore to prevent steric hindrance. This sequence was scrambled as TLQWNQS for control, and was also labeled with Cy5.5, hereafter TLQ*-Cy5.5, Fig. 1B. 3D models show differences in biochemical structures, Fig. 1C,D. Peak absorbance and emission occur in the near-infrared (NIR) spectrum, Fig. 1E,F, where hemoglobin absorption, tissue scattering, and tissue autofluorescence are minimal. The peptides were synthesized with >95% purity by HPLC, and an experimental mass-to-charge ratio (m/z) of 1827.10 was measured using mass spectrometry, which agrees with expected values, Fig. S1A,B.

### Validation of binding with cells ***in vitro***

**Figure 2:**
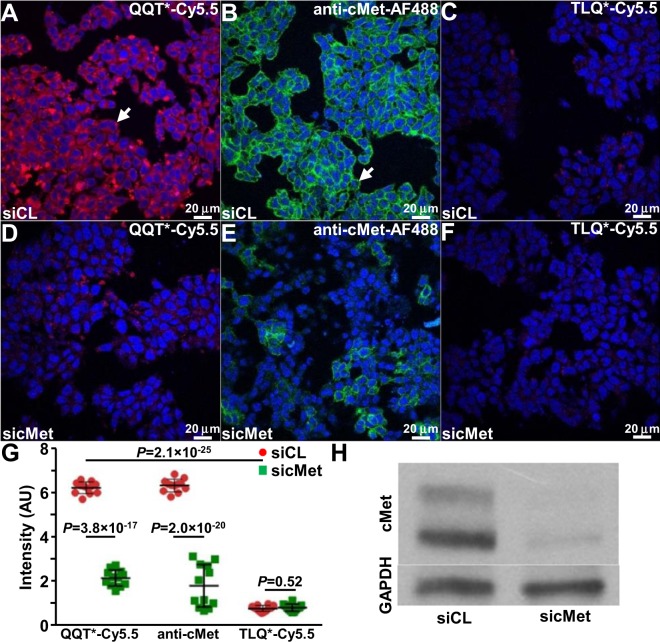
Validation of specific binding with knockdown. (**A**) QQT*-Cy5.5 (red) and (**B**) anti-cMet-AF488 antibody (green) show strong binding to the surface (arrows) of human HT29 CRC cells transfected with siRNA (siCL). **C**) TLQ*-Cy5.5 (red) shows minimal binding. Fluorescence intensities for (**D**) peptide and (**E**) antibody are greatly reduced with knockdown of cMet expression in HT29 cells transfected with sicMet. (**F**) TLQ*-Cy5.5 shows little binding. (**G**) A significant reduction is seen in intensity for QQT*-Cy5.5 and anti-cMet-AF488 for siCL versus sicMet transfected cells (2.9 and 4.1-fold change). TLQ*-Cy5.5 shows a non-significant decrease. The intensity for QQT*-Cy5.5 is significantly greater than that for TLQ*-Cy5.5 (8.1 fold-change). ANOVA models are fit with terms for 6 conditions to log-transformed data. 6 replicate slides are used for each condition with duplicate measures from each slide. The siCL versus sicMet difference for QQT*-Cy5.5 is significantly larger than the same difference for TLQ*-Cy5.5, *P* = 1.4 × 10^−13^. (**H**) Western blot shows cMet expression in cells. The bands are cropped from different parts of the same gel. The original uncropped blots are displayed in Fig. S6A.

siRNA knockdown experiments were performed using human HT29 CRC cells to validate specific binding of QQT*-Cy5.5 to cMet. QQT*-Cy5.5 and anti-cMet-AF488 antibody showed strong binding to the surface of HT29 cells transfected with siCL (control) using confocal microscopy, Fig. 2A,B, while TLQ*-Cy5.5 displayed minimal binding, Fig. 2C. Fluorescence intensities were reduced with HT29 knockdown cells transfected with sicMet, Fig. 2D,E. TLQ*-Cy5.5 revealed little signal, Fig. 2F. Quantified results showed this decrease to be significant, Fig. 2G. Western blot demonstrated effective knockdown of cMet expression, Fig. 2H. Significantly greater fluorescence intensity was observed for binding of QQT*-Cy5.5 and anti-cMet-AF488 to HT29 cells (cMet+) compared with human SW480 CRC cells (cMet−) cells, Fig. S2. Similar results were found for mouse S114 (cMet+) and NIH3T3 (cMet−) cells, Fig. S3.

### Peptide characterization

**Figure 3:**
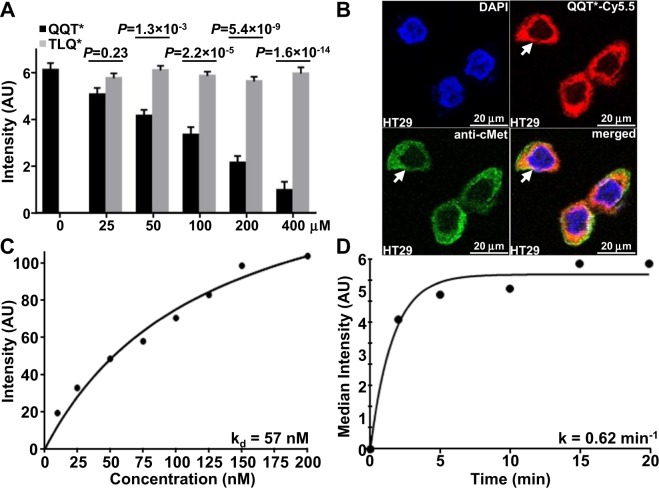
Characterization of peptide binding. **(A**) Binding by QQT*-Cy5.5 to HT29 cells decreases significantly with competition from unlabeled QQT* but not TLQ*. *P*-values compare differences in intensity from QQT*-Cy5.5 after competing with different concentrations of unlabeled QQT* and TLQ*. An ANOVA model with terms for 11 means is fit to log-transformed data with 3 replicate slides per condition. 5 randomly chosen cells per slide are averaged. (**B**) Binding by QQT*-Cy5.5 (red) and anti-cMet-AF488 (green) co-localizes to the surface (arrows) of HT29 cells with a correlation of ρ = 0.73. (**C**) An apparent dissociation constant k_d_ = 57 nM, R^2^ = 0.98, is measured for binding of QQT*-Cy5.5 to HT29 cells. (**D**) An apparent association time constant k = 0.62 min^−1^ (1.6 min), R^2^ = 0.97, is measured for binding of QQT*-Cy5.5 to HT29 cells. Both results are representative of 3 independent experiments.

Specific binding of QQT*-Cy5.5 to cMet was further supported by addition of unlabeled QQT* to compete for binding to HT29 cells. Fluorescence intensities decreased significantly with increasing concentrations of unlabeled QQT* but not with TLQ*, Fig. 3A. These results suggest that the peptide rather than either the linker or fluorophore mediates the binding interaction. By comparison, fluorescence intensities from binding of QQT*-Cy5.5 to HT29 cells did not change with addition of hepatocyte growth factor (HGF), a known ligand for cMet, at concentrations ranging from 0 to 100 ng/mL, Fig. S4A. A pull-down assay showed a strong band from QQT* binding to mouse cMet-ECD by comparison with that for TLQ*, Fig. S4B. Co-localization of QQT*-Cy5.5 and anti-cMet-AF488 binding to the surface of HT29 cells was shown with a correlation of ρ = 0.73, Fig. 3B. An apparent dissociation constant of k_d_ = 57 nM was measured for binding by QQT*-Cy5.5 to HT29 cells using flow cytometry, Fig. 3C. An apparent association time constant of k = 0.62 min^−1^ (1.6 min) was measured to support rapid binding onset, Fig. 3D.

### Effect of peptide on cell signaling

**Figure 4:**
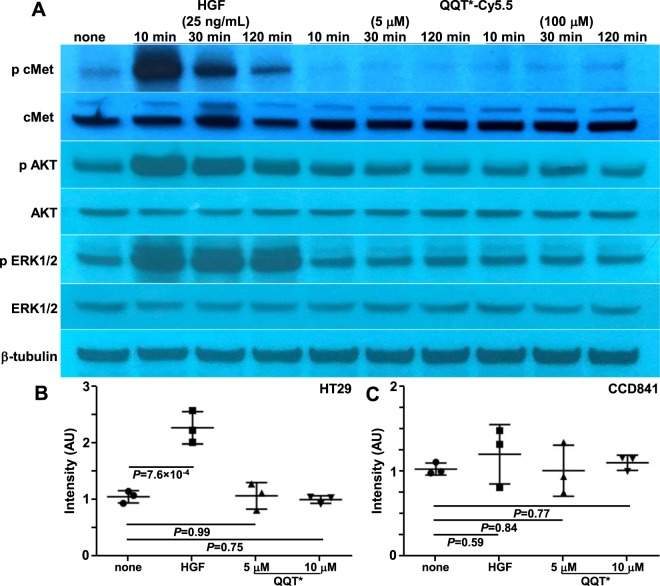
Peptide effect on cell signaling and growth. **(A**) HGF (25 ng/mL) induces phosphorylation of cMet and downstream AKT and Erk1/2 in HT29 cells after 10, 30 and 120 min of incubation from Western blot. No HGF (none) serves as a negative control. Incubation with QQT*-Cy5.5 at either 5 or 100 μM shows no effect on p-cMet expression or downstream AKT and Erk1/2 signaling. β-tubulin is used as a loading control. This group of bands is cropped from different parts of the same gel. The original uncropped blots are displayed in Fig. S6D–H. An alamar blue assay shows increased growth of (**B**) HT29 but not (**C**) CCD841 cells with addition of HGF after 48 hours. No change is seen with either 5 or 100 μM of QQT*-Cy5.5. An ANOVA model with terms for 4 groups is fit to log-transformed data with 3 independent experiments.

No competition was observed between QQT* and HGF to support lack of interactions and effect on downstream signaling, Fig. S4A. These results suggest that the peptide and HGF bind to different sites on the cMet target. Western blots were performed to evaluate markers for activation of downstream cell signaling, Fig. 4A. Incubation of HGF (positive control) with HT29 cells showed strong phosphorylation activity for cMet (p-cMet), downstream AKT (p-AKT), and ERK1/2 (p-ERK1/2). By comparison, addition of QQT*-Cy5.5 at concentrations of 5 and 100 μM resulted in no change in phosphorylation of any substrate. An alamar blue assay showed no effect on growth of HT29 and CCD841 cells with addition of QQT*-Cy5.5 at concentrations of either 5 or 100 μM for 48 hours by comparison with HGF, Fig. 4B,C. CCD841 normal colon cells was used to evaluate the effect of the peptide on the cell phenotype in non-tumor cells.

### ***In vivo*** imaging and macroscopic validation in mouse colon ***ex vivo***

**Figure 5:**
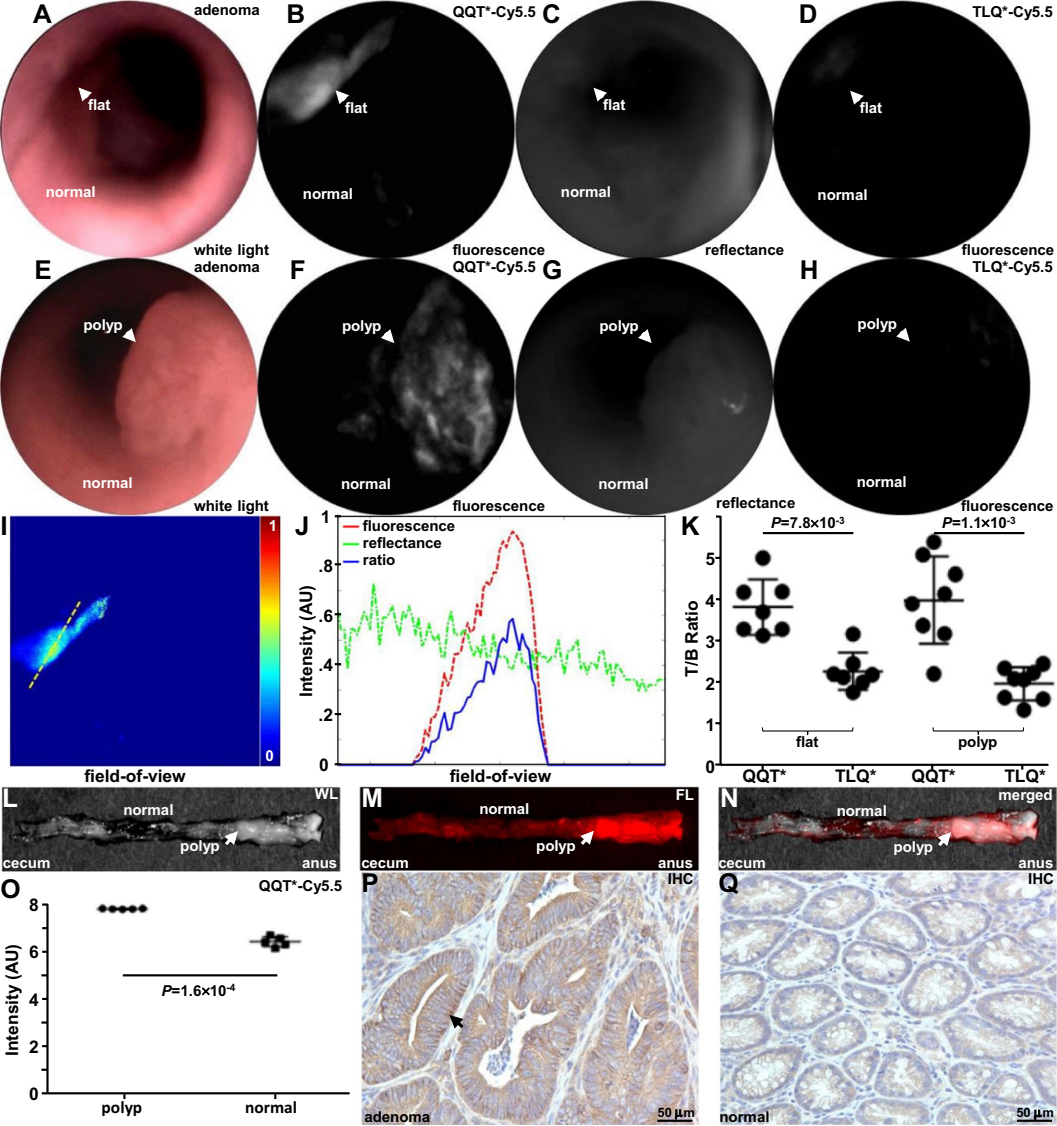
*In vivo* imaging in *CPC;Apc* mice. (**A**) White light image shows no grossly visible lesion (flat). (**B**) NIR fluorescence image after intra-rectal administration of QQT*-Cy5.5 shows increased intensity from the flat lesion (arrow). (**C**) Co-registered reflectance image is acquired from the same lesion. (**D**) Fluorescence image collected using TLQ*-Cy5.5 (control) shows minimal signal. (**E**) White light image of colon shows presence of a polyp (arrow). (**F**) QQT*-Cy5.5 shows increased fluorescence intensity from the polyp (arrow). (**G**) Co-registered reflectance image of polyp is collected. (**H**) TLQ*-Cy5.5 shows minimal signal. (**I**) Ratio of the fluorescence and reflectance images from the flat lesion in (**A**) is shown. (**J**) Fluorescence (red), reflectance (green), and ratio (blue) intensities from the dashed line in (**I**) are shown. (**K**) From n = 8 mice, QQT*-Cy5.5 shows significantly higher mean (±SD) T/B ratio from flat lesions (n = 7) and polyps (n = 8) versus adjacent normal mucosa by paired t-tests on log-transformed data with 1.7 and 2.1-fold change, respectively. (**L**) White light image of excised colon shows numerous polyps (arrow) on exposed mucosal surface. (**M**) Fluorescence image collected *ex vivo* shows increased intensity from polyps after topical administration of QQT*-Cy5.5. (**N**) Merged image. (**O**) From n = 5 mice, the mean fluorescence intensity from adenoma is 2.6-fold higher than that from normal-appearing adjacent normal mucosa by paired t-test on log-transformed data. Immunohistochemistry (IHC) shows higher expression of cMet in (**P**) dysplasia versus (**Q**) normal.

The results of a pull-down assay supported specific binding of QQT*-Cy5.5 to mouse cMet-ECD, Fig. S4B. A rigid small animal endoscope was used to collect *in vivo* images in *CPC;Apc* mice. A representative flat lesion displayed bright fluorescence after intra-rectal administration of QQT*-Cy5.5, while minimal signal was seen when the same lesions were imaged 3 days later using TLQ*-Cy5.5, Fig. 5A–D, Videos S1–S3. Similar results were obtained from a representative polypoid lesion, Fig. 5E–H, Videos S4–S6. A ratio of fluorescence and reflectance images from the flat lesion was determined to correct for differences in distance and geometry over the image field-of-view (FOV) to allow for image intensities to be accurately quantified, Fig. 5I. Fluorescence, reflectance, and ratio values from the dashed line in Fig. 5I were shown, Fig. 5J. Images collected from polyps were processed similarly. The mean T/B ratio was significantly greater for QQT*-Cy5.5 versus TLQ*-Cy5.5 for flat lesions and polyps, Fig. 5K. Imaging was also performed *ex vivo* to validate specific binding by QQT*-Cy5.5 to cMet. The colon was excised and divided longitudinally to expose the mucosal surface. White (WL) and fluorescence (FL) images were shown, Fig. 5L,M. Co-localization at the polyps was seen on the merged image, Fig. 5N. The adenoma borders were clearly seen. The mean fluorescence intensity was significantly greater for polyps versus adjacent normal colonic mucosa, Fig. 5O. Expression of cMet was increased in mouse adenoma versus normal colon using immunohistochemistry (IHC), Fig. 5P,Q.

### Microscopic validation in mouse colon ***ex vivo***

Increased fluorescence from QQT*-Cy5.5 (red) and anti-cMet-AF488 (green) on the surface of dysplastic colonocytes was observed in sections of *CPC;Apc* mouse colon using confocal microscopy, Fig. S5A,B. The merged image showed co-localization of peptide and antibody binding with a correlation of ρ = 0.78, Fig. S5C. Minimal staining was observed for peptide and antibody to normal colonic mucosa, Fig. S5D–F. Quantified results showed significantly greater mean fluorescence intensity for dysplasia versus normal, Fig. S5G. Histology (H&E) for mouse adenoma and normal colon are shown, Fig. S5H,I.

### Validation of cMet expression in human colon

**Figure 6:**
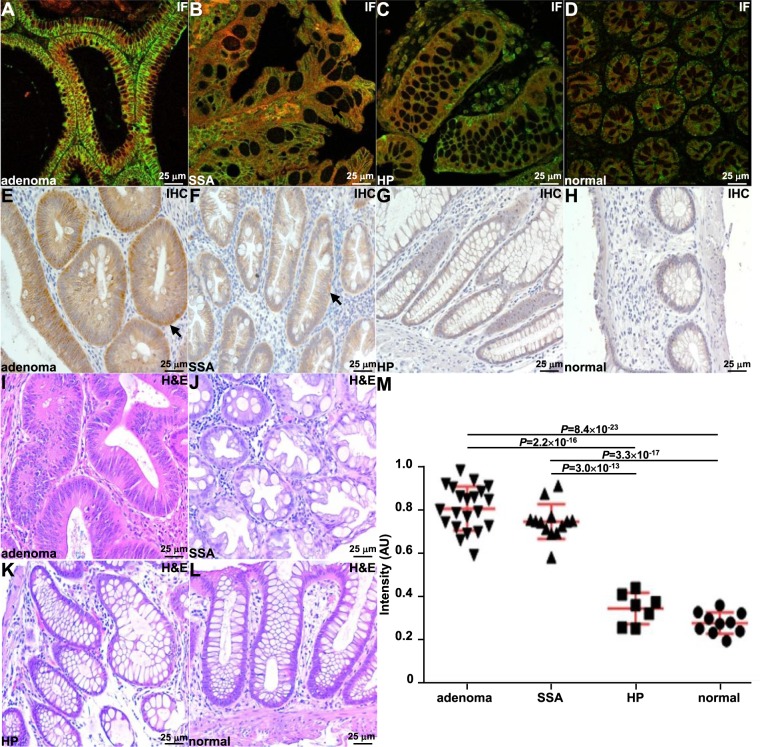
Binding of cMet peptide and antibody to human colon *ex vivo*. Merged images show co-localization of binding for QQT*-Cy5.5 (red) and anti-cMet-AF488 (green) to (**A**) tubular adenoma, (**B**) SSA, (**C**) hyperplastic polyp (HP), and **D**) normal colon with correlation of ρ = 0.82, 0.79, 0.86, and 0.75, respectively. (**E**–**H**) Immunohistochemistry (IHC) performed on representative specimens from same classification supports cMet expression. (**I**–**L**) Representative histology (H&E) is shown. (**M**) The mean fluorescence intensity is significantly higher for adenoma (n = 21) versus normal (n = 10) and HP (n = 7) with 3.0 and 2.4-fold change, respectively, using an ANOVA model with terms for 4 groups on log-transformed data with 3 replicate intensity measures per sample. For SSA (n = 13), the mean fluorescence intensity is also significantly higher than that for normal and HP by 2.8 and 2.2-fold change, respectively. Measurements are the mean of 3 regions of interest (ROI) with dimensions of 20 × 20 μm^2^ on each slide.

Staining of human colon with QQT*-Cy5.5 and anti-cMet-AF488 was evaluated in n = 42 formalin-fixed, paraffin-embedded (FFPE) specimens, including tubular adenoma, sessile serrated adenoma (SSA), hyperplastic polyp (HP), and normal mucosa. Merged fluorescence images showed strong co-localization of peptide and antibody binding for each histological classification, Fig. 6A–D. Immunofluorescence of SSA specimens showed large, dilated crypts with numerous goblet cells to suggest abnormal maturation. Immunohistochemistry was performed to validate cMet expression. Strong staining (2+/3+) was observed for adenoma and SSA while weak staining (0+/1+) was seen for HP and normal, Fig. 6E–H. Representative histology (H&E) was shown, Fig. 6I–L. The mean fluorescence intensity from staining with QQT*-Cy5.5 was significantly greater for either adenoma or SSA versus either HP or normal colon, Fig. 6M. A total of n = 11 SSA lesions were described endoscopically with a flat appearance, and n = 2 were documented to be slightly protruding from colonoscopy reports.

## Discussion

Here, a linear, 7mer peptide specific for cMet was identified using methods of phage display by biopanning against the extracellular domain (ECD) of the purified protein. The peptide was labeled with Cy5.5, a NIR fluorophore, to demonstrate proof-of-concept for *in vivo* imaging as a targeted contrast agent. Specific binding was validated *in vitro* using human CRC cells with knockdown, competition, and co-localization studies. A binding affinity of k_d_ = 57 nM and binding onset of k = 0.62 min^−1^ (1.6 min) were measured. Unlike HGF, peptide binding shows no effect on downstream cell signaling. Fluorescence images collected *in vivo* from flat and grossly visible polyps in the colon of *CPC;Apc* mice showed high T/B ratios. Fluorescence images collected *ex vivo* from human colon specimens supports strong peptide binding to traditional adenomas and SSAs. Significantly less signal was seen with hyperplastic and normal colon. Experiments were controlled with a scrambled peptide.

Current endoscopic methods for early detection of CRC use white light illumination alone, and are not sensitive to flat lesions. Molecular targets are expressed well before structural changes become apparent, such as polyp formation. Peptides developed for overexpressed targets can be used to detect pre-malignant lesions that are flat in appearance and easily missed during colonoscopy with white light illumination alone. The fluorescence images collected *in vivo* from flat pre-malignant lesions in mouse colon illustrate this approach. In our study, n = 7 lesions with non-polypoid morphology were visible only on fluorescence images generated following peptide binding. All of these flat lesions were identified as dysplasia on pathology. The *CPC;Apc* mice used for imaging were genetically engineered to somatically delete an *APC* allele under Cre regulation, and adenomas develop spontaneously in the distal colon^39^. This model is representative of human disease as Apc mutations are found in > 80% of sporadic colorectal cancers^40^. Most SSAs and many sporadic adenomas have a flat appearance during colonoscopy. The serrated and traditional signaling pathways account for >95% of all sporadic CRC’s^41,42^. The remaining ~5% is attributed to genetic syndromes, such as hereditary nonpolyposis colorectal cancer (HNPCC) and familial adenomatous polyposis (FAP)^43,44^.

A 26-mer cyclic peptide specific for cMet has been previously developed. This peptide was labeled with a modified Cy5 fluorophore, and was used to collect clinical images with a fluorescence colonoscope^21^. This peptide was found to have high affinity with a k_d_ = 3 nM. Imaging was performed ~3 hours post-injection to allow time for background to clear. A total of n = 15 patients at high risk for CRC were studied, and n = 101 tubular adenomas were detected with white light illumination alone. An additional n = 22 adenomas were found with fluorescence. The mean T/B ratio was only slightly greater than 1. Our peptide is much smaller in size (7 versus 22 amino acids), and provides less surface area for non-specific interactions that can increase background. Although the binding affinity is not as strong (57 versus 3 nM), it appears to be adequate for *in vivo* use as a diagnostic imaging agent. Immunofluorescence of human sections was performed to support specific binding by this peptide.

Phage display was used previously to identify a different 12-mer peptide by subtractive biopanning against whole S114 cells genetically engineered to overexpress cMet. This peptide was radiolabeled with ^125^I, and nuclear images were collected from mouse xenograft tumors *in vivo*. Cancer-associated activity was seen up to 24 hours post-injection^45^. A binding affinity of k_d_ = 64 nM was measured using cells *in vitro*. This result is comparable with ours. No staining of human tissues was performed to assess for clinical relevance, and the experiments performed were not controlled using an alternate sequence. By comparison, we biopanned a phage display library against purified cMet-ECD protein. This approach minimizes non-specific interactions with non-target proteins expressed on the cell membrane, and should improve binding specificity.

Large molecules, such as antibodies, that have high target affinity are also being utilized for CRC imaging. Bevacizumab is a monoclonal antibody that has been repurposed for imaging by labeling with IRDye800 to detect overexpressed vascular endothelial growth factor A (VEGFA)^46^. This imaging agent was intravenously administered in a total of n = 17 patients with familial adenomatous polyposis (FAP), and n = 51 adenomas were detected. A median T/B ratio = 1.84 was achieved using a dose of 25 mg. Small adenomas with dimensions< 3 mm were detected. Antibodies have long circulation times that lead to increased background. Peptides, by comparison, are much smaller in size and lower in molecular weight. These properties can be developed to overcome many challenges for probe delivery, including irregular microvasculature, heterogeneous uptake, and transport barriers^47–49^. Improved diffusion and extravasation through leaky vessels can result in higher concentrations and deeper penetration^50–52^. Also, peptides have less potential for immunogenicity^53^.

This study demonstrates promise for a small fluorescently-labeled peptide to detect pre-malignant colonic lesions *in vivo*. The reduced size may decrease background, immunogenicity, and cost for mass manufacture. These features can overcome some of the limitations of larger ligands, and are well suited for clinical use in high volume procedures, such as colonoscopy. In this study, the peptides were administered intra-rectally in a mouse model of CRC for convenience. Intravenous administration is needed for adequate distribution throughout all colonic segments. The pharmacokinetics and stability of this peptide in circulation should be characterized. Use of IRDye800 to label the peptide may improve tissue penetration depth and water solubility. Immunofluorescence results using human colon specimens support peptide binding to sporadic adenomas and SSAs. This approach has potential to detect missed pre-malignant lesions that arise from the traditional and serrated signaling pathways.

## Methods

### Ethical approval and informed consent

All experimental procedures were performed in accordance with relevant guidelines and regulations of the University of Michigan. Mice were housed per guidelines of the Unit for Laboratory Animal Medicine (ULAM), and *in vivo* imaging was performed with approval by the University of Michigan Committee on the Use and Care of Animals (UCUCA). All patient reports and human tissues used were deidentified prior to study.

### Cells lines, culture media and chemicals

Human HT29, SW480, CCD841 colorectal cancer cells and mouse NIH 3T3 embryonic fibroblast cells were obtained from the ATCC. S114 cells (NIH 3T3 cells transformed to express cMet) were provided by courtesy of G Vande Woude (Van Andel Institute). McCoy’s 5A Medium (Gibco) was used for HT29 cells. Dulbecco’s Modified Eagle Medium (Gibco) were used for SW480, NIH3T3, and S114 cells. Eagle’s Minimum Essential Medium (Lonza) was used for CCD841. All cells were cultured at 37 °C in 5% CO_2_, and supplemented with 10% fetal bovine serum. Reagents for peptide synthesis were obtained from Anaspec (Anaspec) and AAPPTEC (AAPPTEC) at the highest available grade (>99% purity), and were used without further purification. Solvents and other chemical reagents were purchased (Sigma-Aldrich) unless stated otherwise.

### Peptide specific for cMet

A phage display library of hepapeptides (Ph.D.-7, New England Biolabs) with diversity of ~10^9^ was used to biopan against the extra-cellular domain (ECD) of purified cMet protein (10692-H08H, Sino Biological Inc) for 4 rounds^54^. Candidate phages with the highest enrichment were selected for further evaluation. Reactivity to HT29 cells was assessed using enzyme-linked immunosorbent assay (ELISA). Binding interactions between the candidate peptides and cMet were assessed with non-intact structures 1UX3 and 2UZX using Pepsite software^55^. Peptides were synthesized using standard Fmoc-mediated solid-phase chemistry using an automatic synthesizer (PS3, Protein Technologies Inc)^56^. The C-terminus was labeled with Cy5.5 (Lumiprobe) via a 5 amino acid (GGGSK) linker. Crude peptides were purified with HPLC (Waters) using a C18 column. The final purity was confirmed using an analytical C18 column. The mass-to-charge ratio (m/z) was measured using mass spectrometry (MALDI-TOF, Bruker AutoFlex Speed).

### Spectral measurements

The peptide absorbance spectrum was measured using a UV-Vis spectrophotometer (NanoDrop 2000, Thermo Scientific). Fluorescence emission was collected with a fiber coupled spectrophotometer (Ocean Optics) using a diode pumped solid state laser (Technica Laser Inc) with excitation at λ_ex_ = 671 nm. The spectra were plotted using Origin 6.1 software (OriginLab Corp).

### Confocal fluorescence microscopy

The cells were blocked with 1X PBS plus 2% BSA for 1 hour at 4 °C, and were incubated with 5 μM of peptide for 10 min at room temperature (RT) in the dark, washed 3×, and fixed with 4% PFA for 5 min, washed with 1X PBS, and then mounted on glass slides with ProLong Gold reagent containing DAPI (Invitrogen). A 1:3000 dilution of primary monoclonal rabbit anti-cMet antibody (#8198, Cell Signaling Technology) was incubated with the cells *in vitro* as a positive control. Afterwards, the cells were incubated with a 1:500 dilution of AF488-labeled secondary goat ant-rabbit immunoglobulin G antibody (#A-11029, Life Technologies), and then mounted on glass slides with ProLong Gold reagent containing DAPI. Confocal fluorescence images were collected using a 63X oil-immersion objective (Leica SP5 Inverted 2-Photon FLIM confocal). Fluorescence intensities were quantified using custom Matlab software (Mathworks).

### Downregulation of cMet with siRNA

Expression of cMet by HT29 cells was knocked down using siRNA (siRNA1, SASI_Hs01_00133002, Sigma). MISSION^®^ siRNA Universal Negative Control (SIC001, Sigma) was used for control. Cells were transfected with Lipofectamine 2000 (11668027, Invitrogen) per manufacturer instructions, and incubated with 5 μM of peptide for 5 min. A 1:3000 dilution of primary monoclonal rabbit anti-cMet antibody (Cell Signaling Technology, #8198) was used for positive control.

### Competition for peptide binding

Specific peptide binding to HT29 cells was evaluated using competitive inhibition with unlabeled peptide and recombinant human hepatocyte growth factor (HGF, 294-HG-005, R&D). Unlabeled peptides at 0, 25, 50, 100, 200 and 400 μM were first added and then incubated with 5 μM of Cy5.5-labeled peptides. HGF was added in quantities of 0, 5, 10, 25, 50 and 100 ng/mL. The cells were fixed with 4% PFA, and mounted with ProLong Gold reagent containing DAPI.

### Peptide characterization

The apparent dissociation constant k_d_ was measured to estimate the peptide binding affinity to HT29 cells. HT29 cells were incubated with peptide at concentrations of 0, 10, 25, 50, 75, 100, 125, 150, and 200 nM for 1 hour at 4 °C. The cells were analyzed using flow cytometry (FACS Canto; BD Biosciences). Sample means were used to calculate k_d_ using a nonlinear regression analysis with Origin 6.1 software (OriginLab). The parameter k_d_ = 1/k_a_ was calculated by performing a least squares fit of the data to the non-linear equation I[X] = (I_0_ + I_max_k_a_[X])/(I_0_ + k_a_[X]), where I_0_ and I_max_ are the initial and maximum fluorescence intensities, corresponding to no peptide and at saturation, respectively^57^.

The time scale for peptide binding to HT29 cells was assessed by estimating the apparent association time constant k. HT29 cells were incubated with 5 μM of Cy5.5-labeled peptides for time intervals ranging between 0 and 20 min at 4 °C. The cells were fixed with 4% PFA for 30 min at 4 °C before analyzing with flow cytometry. The rate constant was calculated by fitting the data to a first-order kinetics model y(t) = I_max_ [1− exp(−kt)], where I_max_ is the maximum value, using Prism 5.0 software (GraphPad)^58^.

Interactions between peptide and mouse cMet were evaluated using a pull-down assay^59^. Peptides were immobilized on EHS active beads (17-0906-01, GE), and incubated with purified mouse cMet-ECD protein (50622-M08H, Sino Biological). After washing, bound proteins were detected by Western blot.

### Effect of peptide on cell signaling

HT29 cells were treated with serum free medium overnight for starvation before incubation with either HGF or peptides. HGF was added at concentrations of 25 ng/mL for 10, 30, and 120 min in separate wells. Peptides are added at concentrations of 5 and 100 μM for 10, 30, and 120 min. Anti-cMet antibody (#8198, Cell Signaling), phospho-cMet (Tyr1234/1235) antibody (#3077, Cell Signaling), anti-AKT (#4691, Cell Signaling), anti-phospho-AKT (#9271, Cell Signaling), anti-ERK1/2 (#ab17942, Abcam,), anti-phospho-ERK1/2 (#ab50011, Abcam), and anti-tubulin (#32-2600, Invitrogen) were used per manufacturer’s instructions.

An alamar blue assay was performed using HT29 and CCD841 cells. After culturing in serum free media overnight, ~3 × 10^3^ cells were seeded per well in serum free media in 96 well plates at a final volume of 100 µL per well. The cells were incubated with either HGF (25 ng/mL) or peptide (5 and 10 μM) at 37 °C for 48 hours. Alamar blue reagent (10 µL) was added in amounts equal to 10% of the volume in the well, and incubated at 37 °C for 4 hours. Fluorescence with excitation at λ_ex_ = 530–560 nm, and emission at λ_ex_ = 590 nm was measured.

### ***In vivo*** imaging and macroscopic validation in mouse colon ***ex vivo***

*CPC;Apc* mice were used for *in vivo* imaging. This mouse was genetically engineered to sporadically delete an adenomatous polyposis coli (*APC*) allele under control of a Cdx2 promoter (CDX2P-9.5NLS-Cre) to spontaneously form either flat or polypoid adenomas in the distal colon^39^. Prior to imaging, the mice were fasted for 4–6 hours. Anesthesia was induced and maintained via a nose cone with inhaled isoflurane mixed with oxygen at concentrations of 2–4% at a flow rate of 0.5 L/min. A rigid small animal endoscope (Karl Storz Veterinary Endoscopy) was inserted into the rectum^60^. White-light illumination was used first to identify the presence of adenomas. Peptides (100 μM, 1.5 mL) were delivered intra-rectally through the instrument channel. After 5 min for incubation, the colon was rinsed with warm tap water 3X to remove stool and debris prior to image collection. After 3 days for the signal from the target peptide to clear, the same mice were imaged using the control peptide. A ratio of the fluorescence and reflectance images was determined to correct for differences in distance and geometry over the image field-of-view (FOV)^33^. A total of 3 independent regions with dimensions of 20 × 20 μm^2^ were identified randomly from the location of the adenoma (target) and from adjacent normal colonic mucosa (background). The mean fluorescence intensity was used to calculate the target-to-background (T/B) ratios. Images were processed and analyzed using custom software in Matlab (Mathworks)^34^.

After completion of *in vivo* imaging, the mice were euthanized. The colon was resected and divided longitudinally. A NIR whole body imaging system (Pearl®, LI-COR Biosciences) was used to collect fluorescence *ex vivo*. A FOV of 16.8 × 12 cm^2^ was used to collect images with 85 μm resolution using excitation at λ_ex_ = 685 nm and emission centered at λ_em_ = 720 nm. Images were analyzed using custom software (Image Studio, Li-Cor Biosciences). Regions of adjacent normal colon with area equal to that of adenomas was used for background.

### Microscopic validation in mouse colon ***ex vivo***

Serial formalin-fixed sections of mouse colon were cut with 10 μm thickness and incubated overnight with 1:100 dilution of monoclonal rabbit anti-cMet antibody (EP1454Y, Abcam) at 4 °C. A 1:200 dilution of secondary goat anti-rabbit antibody (ab150077, Abcam) was applied. Controls were prepared using same method without primary anti-cMet antibody.

### Validation of cMet expression in human colon

Formalin-fixed, paraffin-embedded (FFPE) sections of human specimens, including tubular adenomas (n = 21), sessile serrated adenomas (n = 13), hyperplastic polyps (n = 7), and normal colonic mucosa (n = 10) were obtained from the archived tissue bank in the Department of Pathology. The sections were incubated with 5 μM of peptide followed by a 1:200 dilution of anti-cMet primary antibody (#8198, Cell Signaling Technology) and a 1:500 dilution of AF488-labeled goat anti- rabbit secondary antibody (ab150077, Abcam). The mean fluorescence intensity from each image was measured from 3 boxes with dimensions of 20 × 20 μm^2^ placed completely within colonic epithelium using custom Matlab software. Regions of saturated image intensities were avoided.

## Supplementary information

White light imaging of flat lesion in mouse colon.

Fluorescence imaging of flat lesion in mouse colon with cMet peptide.

Fluorescence imaging of flat lesion in mouse colon with control peptide.

White light imaging of polyp in mouse colon.

Fluorescence imaging of polyp in mouse colon with cMet peptide.

Fluorescence imaging of polyp in mouse colon with control peptide.

Detection of colonic neoplasia in vivo using near-infrared-labeled peptide targeting cMet

## Data Availability

The datasets generated are available by request.
