# Supplementary material for: Detection of colonic neoplasia in vivo using near-infrared-labeled peptide targeting cMet
Source: Sci Rep. 2019 Nov 29;9:17917. doi: 10.1038/s41598-019-54385-7 (PMC6884535; doi:10.1038/s41598-019-54385-7)
Supplement: Supplementary file 7 — Detection of colonic neoplasia in vivo using near-infrared-labeled peptide targeting cMet [file 41598_2019_54385_MOESM7_ESM.pdf]

Detection of colonic neoplasia in vivo using near-infrared-labeled peptide targeting cMet

Xiaoli Wu[1], Juan Zhou[1], Fa Wang[1], Xiaoqing Meng[1], Jing Chen[1], Tse-Shao Chang[5], Miki Lee[1], Gaoming Li[1], Xue Li[1], Henry D. Appelman[2], Rork Kuick[3], Thomas D. Wang[1,4,5*]

[1]Division of Gastroenterology, Department of Internal Medicine, University of Michigan, Ann Arbor, Michigan, USA;

[2]Department of Pathology, University of Michigan, Ann Arbor, Michigan, USA;

[3]Department of Biostatistics, University of Michigan, Ann Arbor, Michigan, USA;

[4]Department of Biomedical Engineering, University of Michigan, Ann Arbor, Michigan, USA;

[5]Department of Mechanical Engineering, University of Michigan, Ann Arbor, Michigan, USA.

Corresponding author:

Thomas D. Wang, M.D., Ph.D.

Professor of Internal Medicine, Biomedical Engineering, and Mechanical Engineering

Division of Gastroenterology, University of Michigan

109 Zina Pitcher Pl. BSRB 1522

Ann Arbor, MI 48109-2200

Office: (734) 936-1228

Fax: (734) 647-7950

Email: thomaswa@umich.edu

Supplementary Figures

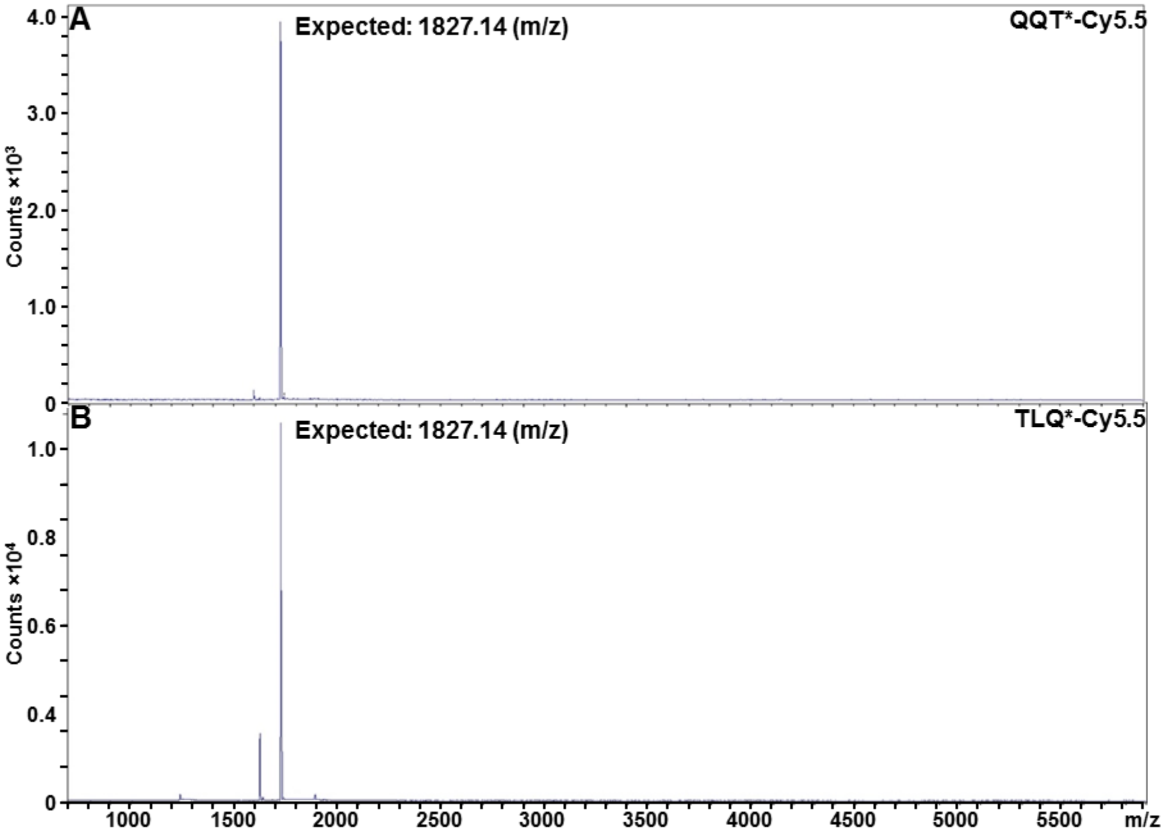

Fig. S1 – Mass spectrometry of Cy5.5-labeled peptides. Experimental mass-to-charge ratios (m/z) for **A**) QQT*-Cy5.5 and **B**) TLQ*-Cy5.5 were found to be 1827.10, which agree with the expected value of 1827.14.

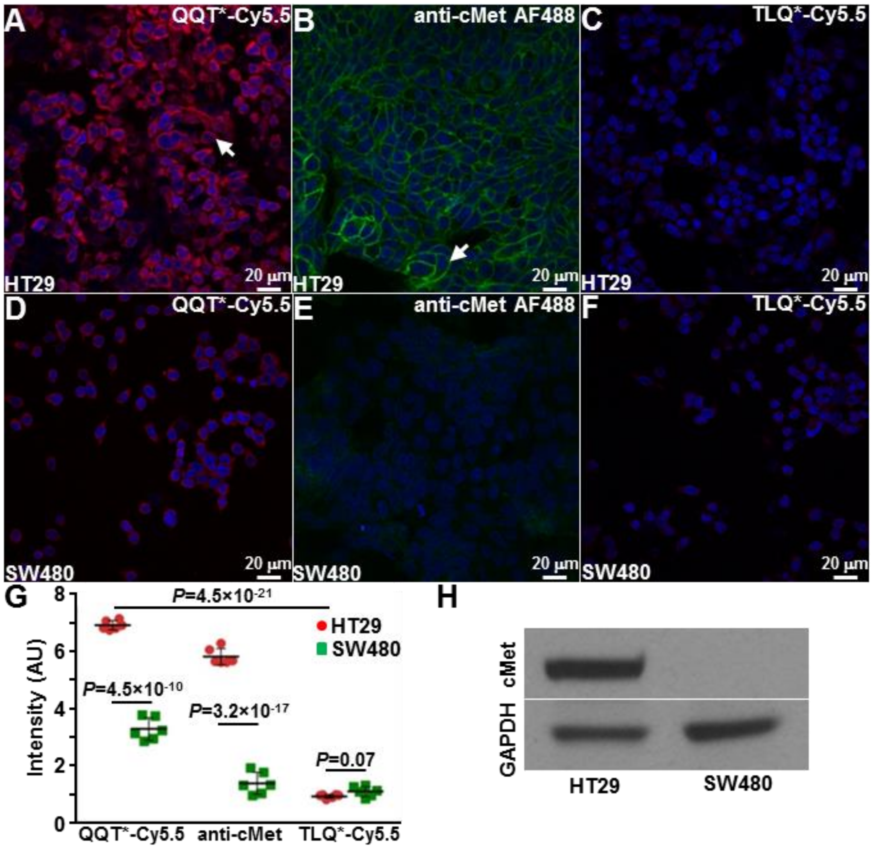

Fig. **S2 – Peptide validation with human CRC cells in vitro**. **A**) QQT*-Cy5.5 (red) and **B**) anti-cMet-AF488 (green) show strong binding to the surface (arrow) of HT29 cells (cMet+). **C**) Control peptide TLQ*-Cy5.5 (red) shows minimal binding. By comparison, signal is reduced with **D**) peptide and **E**) antibody to SW480 cells (cMet-). **F**) TLQ*-Cy5.5 (red) shows minimal binding. **G**) The mean intensities are significantly greater for QQT*-Cy5.5 and anti-cMet-AF488 for HT29 versus SW480 cells with 2.1 and 4.3-fold change, respectively. TLQ*-Cy5.5 shows a non-significant decrease with 0.85-fold change. The mean intensity was significantly greater for QQT*-Cy5.5 versus TLQ*-Cy5.5 with 7.5 fold-change. The HT29 versus SW480 difference was significantly higher for QQT*-Cy5.5 than the same difference for TQL*-Cy5.5 ($P=1.3\times10^{-8}$). An ANOVA model was fit to log-transformed data with terms for 6 conditions. There were 6 replicate slides per condition and 10 randomly chosen cells measured per slide. **H**)

Western blot shows cMet expression for each cell. This group of bands was cropped from different parts of the same gel. The original uncropped blots are displayed in Fig. S6B.

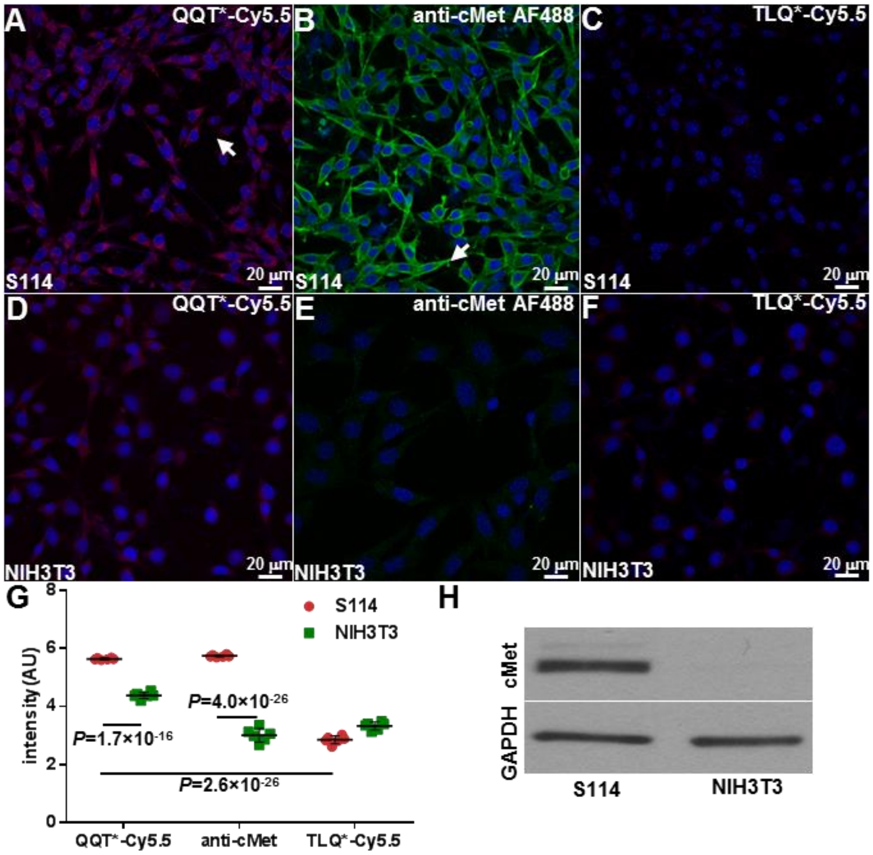

Fig. **S3 – Peptide validation with mouse cells in vitro**. **A**) QQT*-Cy5.5 (red) and **B**) anti-cMet-AF488 (green) show strong binding to the surface (arrow) of S114 cells (cMet+). **C**) TLQ*-Cy5.5 (red) shows minimal binding. By comparison, signal is reduced for **D**) peptide and **E**) antibody to NIH3T3 cells (cMet-). **F**) TLQ*-Cy5.5 (red) shows nominal binding. **G**) The mean intensity was significantly greater for QQT*-Cy5.5 and anti-cMet-AF488 with S114 versus NIH3T3 cells with 2.4 and 6.7-fold change, respectively. TLQ*-Cy5.5 shows a non-significant increase. The mean intensity was significantly greater for QQT*-Cy5.5 versus TLQ*-Cy5.5 with 7.0-fold change. The S114 versus NIH3T3 difference was significantly higher for QQT*-Cy5.5 than the same difference for TQL*-Cy5.5 ($P=1.3\times10^{-8}$). An ANOVA model was fit to log-transformed data with terms for 6 conditions. There were 6 replicate slide per condition and 10 cells measured per slide. **H**) Western blot shows cMet expression for each cell. This group

of bands was cropped from different parts of the same gel. The original uncropped blots are displayed in Fig. S6C.

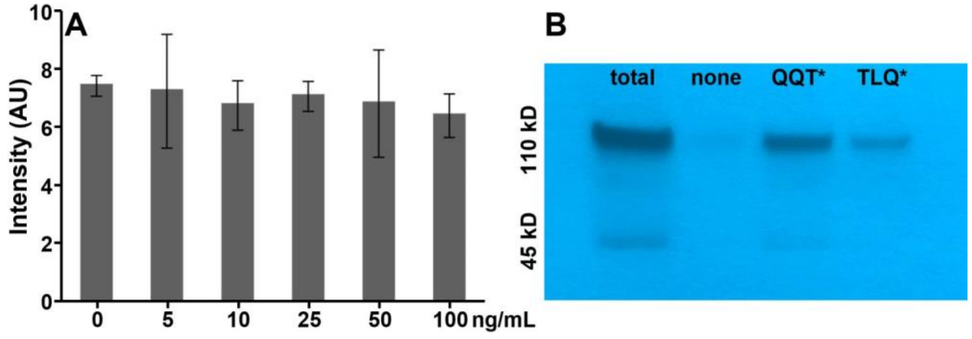

Fig. **S4** – **Peptide characterization**. **A**) Binding by QQT*-Cy5.5 to HT29 cells showed no change with addition of HGF using confocal microscopy. **B**) A strong band is seen for QQT* binding to mouse cMet-ECD versus that for TLQ* from pull-down assay. Key: total – 20 μg mouse cMet-ECD with no EHS beads; none – EHS beads with no peptide; QQT* – target peptide immobilized on EHS beads; TLQ* – control peptide immobilized on EHS beads.

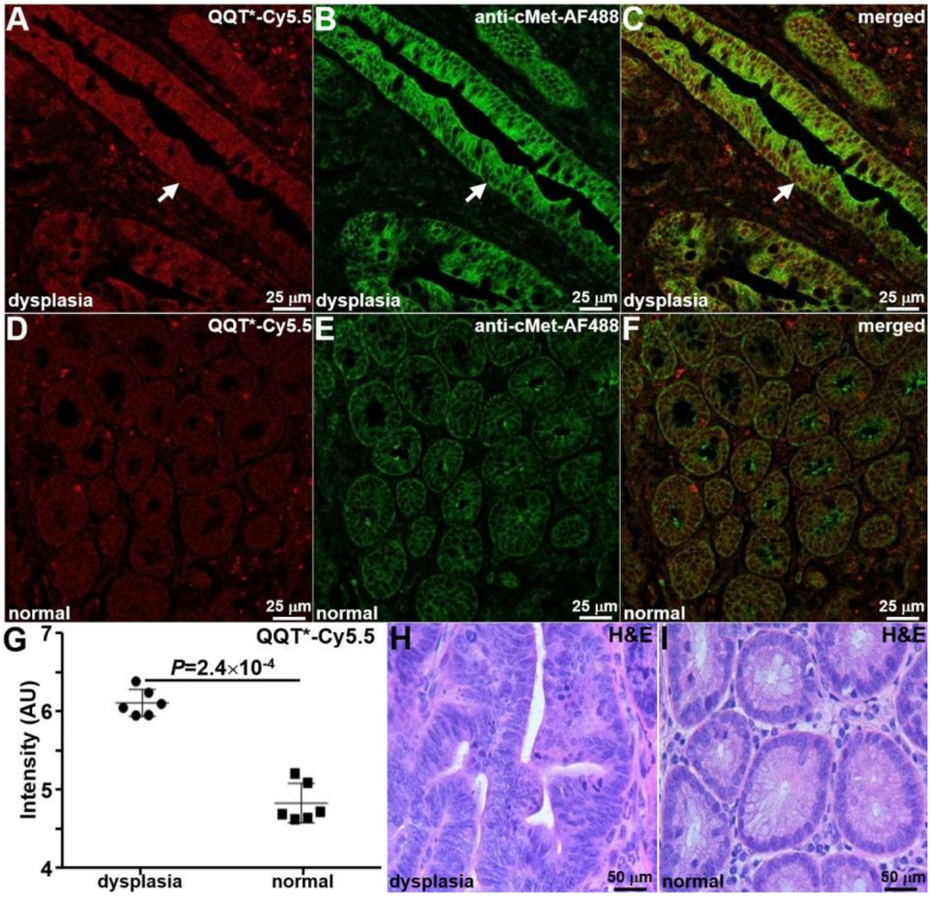

Fig. **S5** – **Microscopic validation of peptide binding in mouse colon ex vivo**. **A**) QQT*-Cy5.5 (red) and **B**) anti-cMet-AF488 (green) binding co-localize with correlation of ρ = 0.78 on the **C**) merged image. **D**-**F**) Minimal signal is observed for normal mucosa. **G**) The mean (±SD) fluorescence intensity was significantly higher for adenoma (n = 6) than for normal (n = 6) with 1.3-fold change by paired t-test on log-transformed data. **H**,**I**) Histology (H&E) is shown.

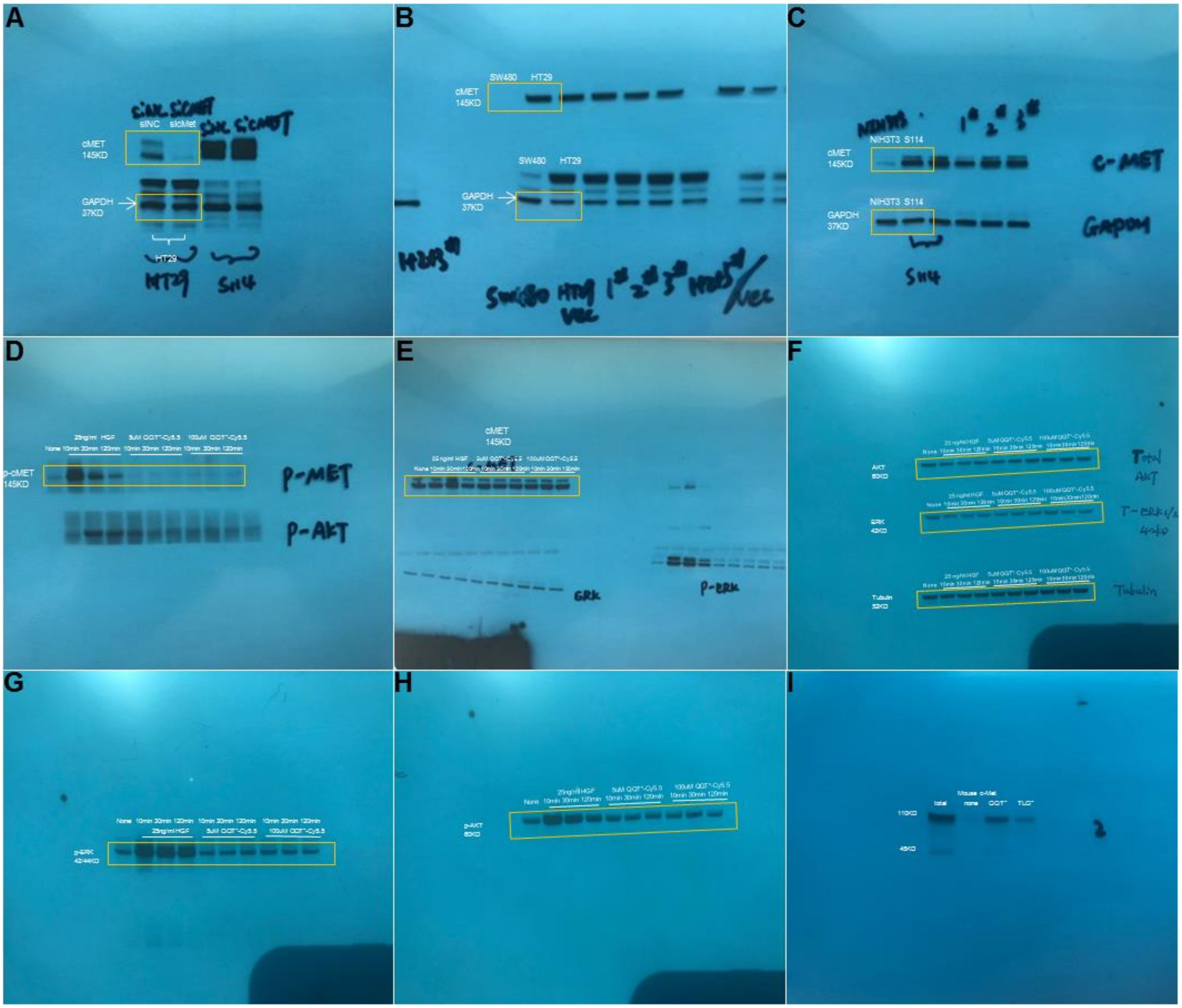

Fig. **S6** – **Original Western blots**. Uncropped gels are shown for **A**) cMet knockdown with siRNA in HT29 cells (Fig. 2H), **B**) cMet expression in HT29 and SW480 cells (Fig. S2H), **C**) cMet expression in S114 and NIH3T3 cells (Fig. S3H), and **D**-**H**) detection of downstream cMet signaling in HT29 cells (Fig. 4) treated with HGF (25 ng/mL) or QQT*-Cy5.5 (5 or 10 μM), **I**) binding of QQT* to mouse cMet-ECD (Fig. S4). Note: all of the blots are from the same gel, and were developed on different films.

Supplementary videos

Video S1 – White light imaging of flat lesion in mouse colon.

Video S2 – Fluorescence imaging of flat lesion in mouse colon with cMet peptide.

Video S3 – Fluorescence imaging of flat lesion in mouse colon with control peptide.

Video S4 – White light imaging of polyp in mouse colon.

Video S5 – Fluorescence imaging of polyp in mouse colon with cMet peptide.

Video S6 – Fluorescence imaging of polyp in mouse colon with control peptide.
